# The Efficacy of Intraoperative Passive Language Mapping for Glioma Surgery: A Case Report

**DOI:** 10.3389/fneur.2021.652401

**Published:** 2021-08-02

**Authors:** Kohei Kanaya, Takumi Mitsuhashi, Takafumi Kiuchi, Sumio Kobayashi

**Affiliations:** ^1^Department of Neurosurgery, Shinshu University School of Medicine, Matsumoto, Japan; ^2^Department of Neurosurgery, Iida Municipal Hospital, Nagano, Japan; ^3^Department of Neurosurgery, Juntendo University, Tokyo, Japan

**Keywords:** cortiq, awake craniotomy, intraoperative passive language mapping, real-time, high-gamma activity, glioma

## Abstract

**Background:** Recently, electrocorticographic (ECoG) studies have emphasized the importance of gamma band-based functional mapping in the presurgical localization of the eloquent cortex. Passive functional mapping using ECoG signals provides a reliable method for identifying receptive language areas without many of the risks and limitations associated with electrical cortical stimulation. We report a surgical case of left temporal malignant glioma with intraoperative passive language mapping.

**Case Description:** A 78-year-old woman was diagnosed with left temporal glioma with inspection of her language difficulty. MRI showed a left temporal tumor measuring 74.6 × 50.0 × 51.5 mm in size. Real-time CortiQ-based mapping using high-gamma activity by word-listening and story-listening tasks was performed. Significant listening task-evoked high gamma activities were detected in 5 channels in the superior temporal gyrus and one channel in the middle temporal gyrus. The tumor was grossly removed except for the region corresponding to listening task-evoked high gamma activities. Postoperatively, the patient's symptoms of language comprehension difficulty improved, and no new neurological deficits were observed.

**Conclusion:** Intraoperative passive language mapping was successfully performed, and the patient's language function was well-preserved. Intraoperative passive language mapping, which is applicable in a short time and under general anesthesia, can be an important tool for detecting language areas.

## Introduction

Electrical cortical stimulation (ECS) mapping remains the gold standard for presurgical localization of brain function (1). Awake craniotomy is the only established method to intraoperatively verify the preservation of language function (2); however, precise functional mapping is still difficult in patients with language deficits (3). ECS has shortcomings, as it is time-consuming and may evoke after-discharges or seizures (4).

Recently, electrocorticographic (ECoG) studies have emphasized the importance of gamma band-based functional mapping in the presurgical localization of the eloquent cortex (5). Passive ECoG-based mapping is set to become one of the most important novel tools in presurgical functional mapping (4).

We report an elderly patient with left temporal glioma evaluated with intraoperative passive language mapping. Tumor removal was successfully performed, preserving her language function.

## Case Description

### Initial Presentation

**Figure 1:**
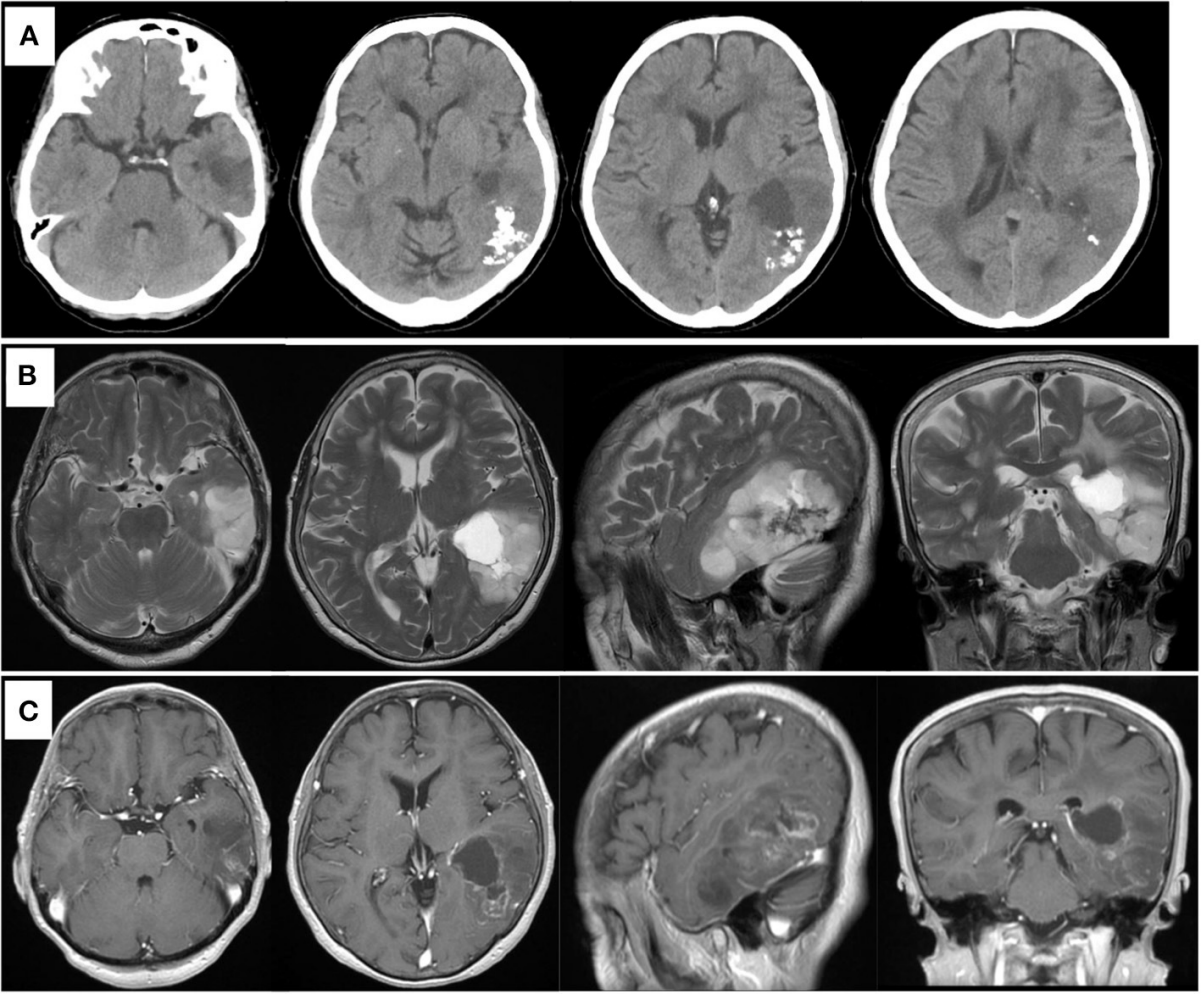
Preoperative computed tomography showing calcified and cystic lesions in the left temporal lobe **(A)**. MR images showing high intensity in the left temporal lobe on T2-weighted image **(B)** and heterogeneous enhanced lesion on gadolinium-enhanced T1- weighted image **(C)**.

A previously healthy 78-year-old woman noticed a worsening of language difficulty and visited our hospital. CT showed a calcified lesion on the left temporal lobe (Figure 1A), and MRI revealed an intra-axial tumor showing high T2 signal and heterogeneous enhancement and a cystic component located medially (Figures 1B,C). The tumor was located in the inferior temporal and middle temporal regions and the fusiform gyrus and 74.6 × 50.0 × 51.5 mm (AP × LR × HT) in size. Preoperative fMRI could not show the Wernicke area clearly because of the large tumor and perifocal edema. Preoperatively, she had fluent aphasia, naming disorder, and difficulty in language comprehension.

Awake craniotomy was abandoned as a possibility considering her aphasia and age. Tumor removal with passive language mapping under general anesthesia was planned to remove as much of the tumor as possible to preserve her language function.

### Intraoperative Passive Language Mapping and Surgery

**Figure 2:**
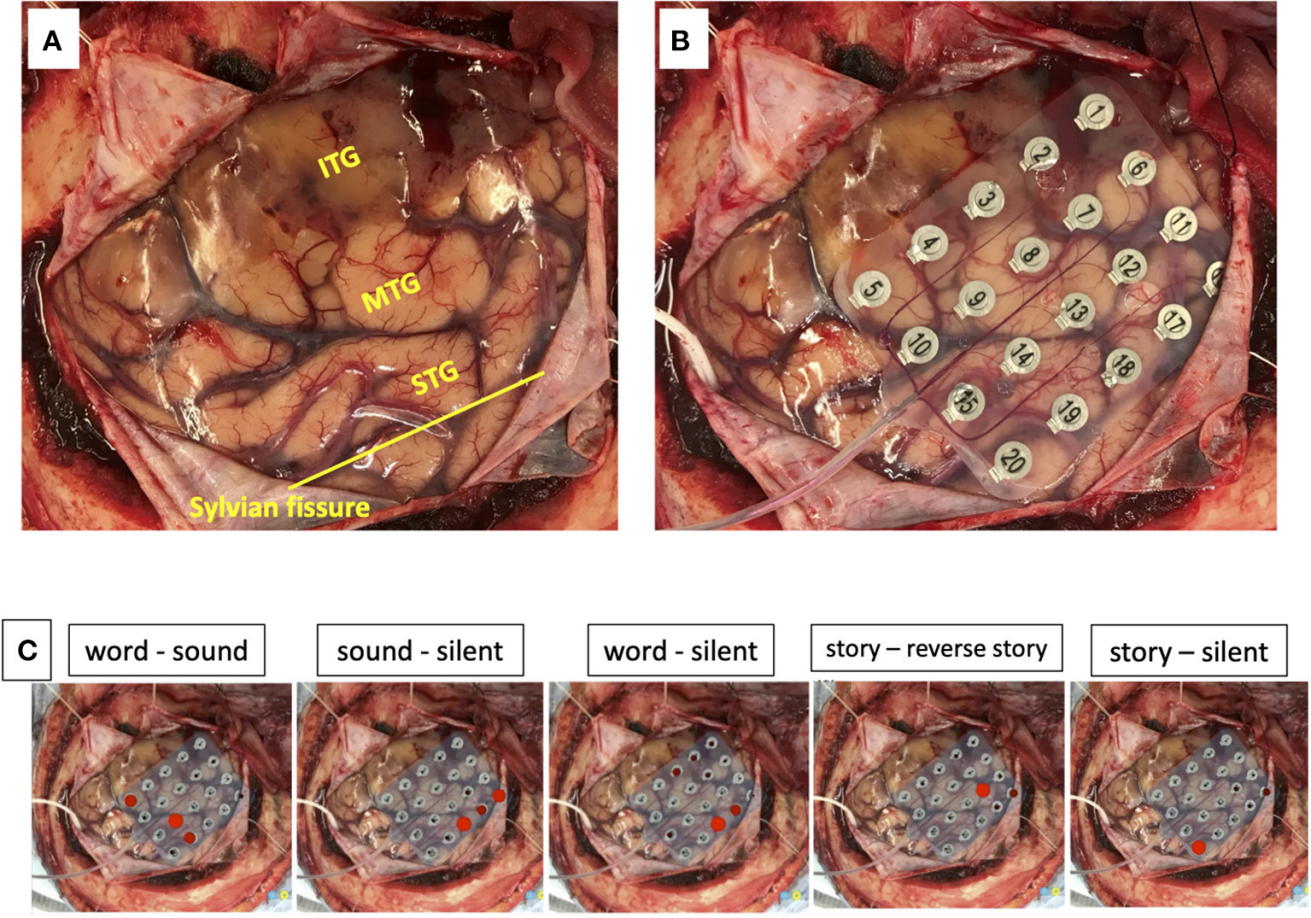
The exposed brain surface **(A)** and a 20-channel ECoG grid placement **(B)** before resection. Red bubble meaning significant listening task-evoked high gamma activities in each task during passive language mapping **(C)**. STG, superior temporal gyrus; MTG, middle temporal gyrus; ITG, inferior temporal gyrus.

**Figure 3:**
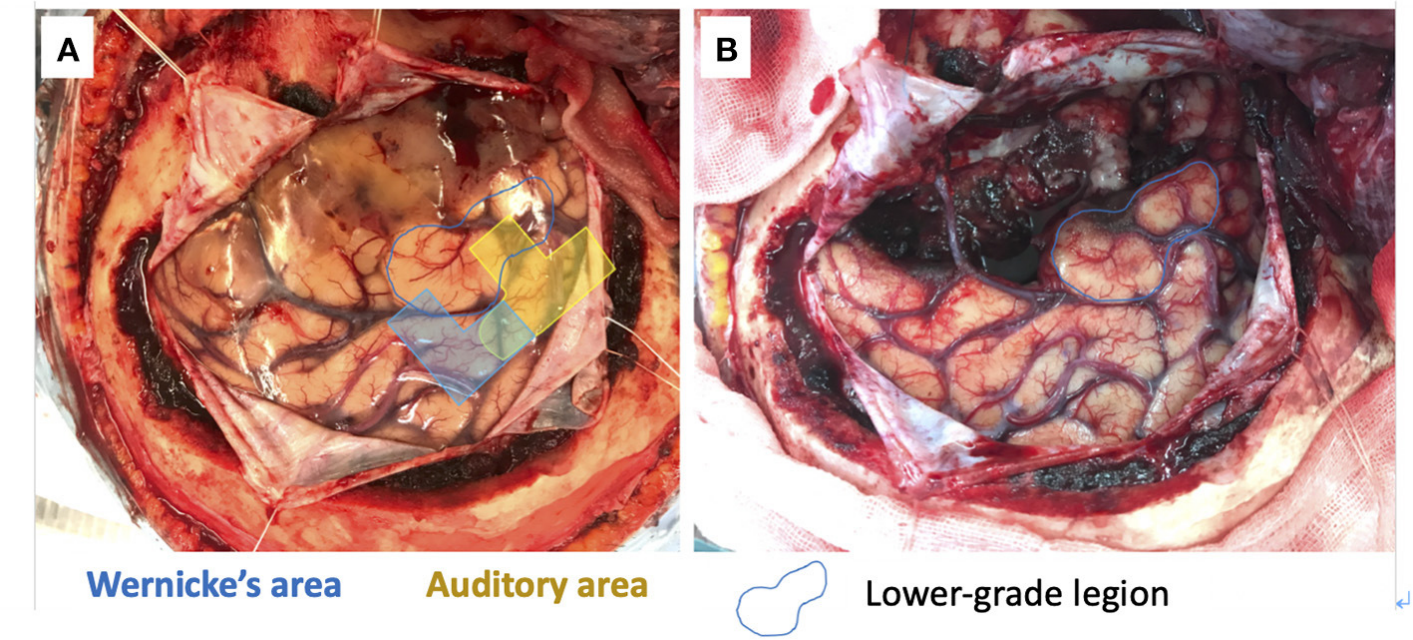
5 channels in the superior temporal gyrus and one channel in the middle temporal gyrus meaning the auditory (yellow) and Wernicke (blue) areas **(A)**. The gross tumor removal except for the lower-grade lesion in the middle temporal gyrus (blue circle) **(B)**.

We used a 20-channel ECoG grid with a 3-mm electrode diameter and a 10-mm interelectrode distance (Unique Medical), together with 4-channel strip electrodes. The exposed brain surface and grid were captured by a digital camera before resection (Figures 2A,B). Real-time CortiQ-based mapping (g.tec medication engineering GmbH, Austria) using high-gamma activity by word-listening (e.g., moon, book, flog, cake, etc.,) and Japanese story-listening tasks was performed. The listening tasks were delivered without sound for 20 s (rest phase) and then delivered sounds for 20 s (active phase). The active phase was composed of meaning and meaningless sounds. The rest and active phases were repeated alternately six times. The sampling rate was 1,200 Hz with a g.HIamp (g.tec medical engineering GmbH, Austria). The analysis was performed by MATLAB 2015a at 60–90 and 110–140 Hz, and over 25% task/rest band power was defined as significant. Anesthesia was maintained with propofol, muscle relaxant, and fentanyl. A bispectral index (BIS) monitor was used to assess the depth of anesthesia and it was maintained with a high BIS level (70–90) during passive language mapping. Significant listening task-evoked high gamma activities were detected (Figure 2C). We analyzed 5 channels in the superior temporal gyrus and one channel in the middle temporal gyrus that were most meaningful for the auditory and Wernicke areas (Figure 3A). The tumor was totally removed except for the region corresponding to the listening task-evoked high gamma activities in the middle temporal gyrus and was suspected to be a lower-grade glioma (Figure 3B).

### Postoperative Course

**Figure 4:**
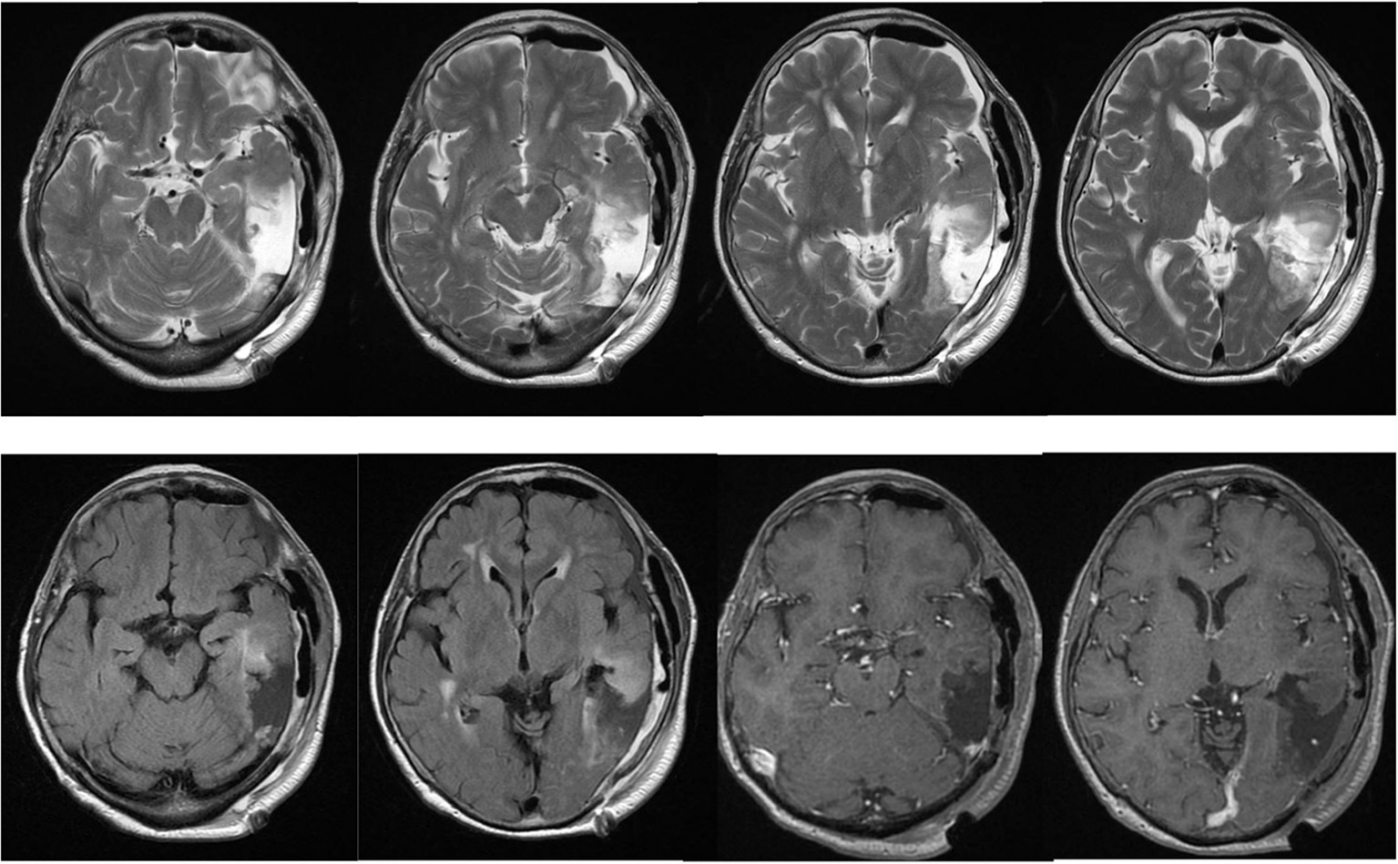
Postoperative MRI showing gross total removal of the tumor.

The patient's postoperative course was uneventful, and her symptoms of language comprehension difficulty improved, although, her naming disorder was unchanged. Postoperative MRI showed gross total resection of the enhancing component (Figure 4). A diagnosis of anaplastic oligodendroglioma (WHO grade 3) was made, and postoperative irradiation and chemotherapy (temozolomide) were performed. She was followed for 1 year without any new neurological deficits or radiological signs of recurrence.

## Discussion

ECS has been the gold standard for functional mapping for decades (4). It is widely accepted that the application of conventional ECS methods produces specific and reliable outcomes at defined sites and that neurosurgical resective strategies guided by this method can eliminate or minimize sensorimotor and linguistic postoperative deficits (6, 7). Awake craniotomy is usually performed in patients aged 15–65 years, although, awake craniotomy is not only specified by age (8). Recently, there have been some reports that awake craniotomy can be successfully performed for elderly and younger patients (9–11); however, the procedure can be difficult to apply for patients with severe aphasia, intellectual disability, or psychiatric disorders, and elderly and younger patients who cannot tolerate awake craniotomy (12, 13). Furthermore, ECS has noteworthy limitations that include substantial time requirements (14, 15) and an increased risk for induced pathological brain activity, such as after-discharges or seizures (16, 17).

Gamma oscillation may be induced by synchronized activity of γ-aminobutyric acidergic (GABAergic) interneurons in the population network playing an important role in controlling the timing of action potential generation in pyramidal cells (18–21). Several researches have revealed that gamma-band power is positively correlated with neuronal firing rates (22–24).

Tamura et al. reported that the dynamics of oscillatory neuronal activity on ECoG have been proposed as potential neurophysiological indicators. Among these oscillatory changes, augmentation of high gamma activity from ~60 to 140 Hz was assumed to reflect localized cortical processing. Therefore, high gamma activity may be a strong candidate for an index of local cortical activation, and passive language mapping has possibilities of detecting physiological mechanisms related to language in the human brain (3).

Swift et al. reported that the qualitative comparison between cortical sites identified by ECoG and ECS shows a high concordance. However, the authors emphasized that it is important to evaluate language function by not only ECoG mapping but also other modalities, such as ECS, although, passive functional mapping using ECoG signals provides a fast, robust, and reliable method for identifying receptive language areas without many of the risks and limitations associated with ECS (4).

Wang et al. reported that electrode sites showing task-related high-gamma augmentation were spatially concordant with, but more extensive than, the language areas suggested by gold standard electrical stimulation mapping (25). Furthermore, Asano and Gotman reported that task-related high-gamma augmentation may be more extensive than electrical stimulation in localizing the cortex participating in language. As a result of this increased sensitivity, task-related high-gamma augmentation could involve a more extensive area than the region that, if removed, would result in clinically noteworthy language deficits (26). Therefore, we speculated that the possibility of postoperative language functional preservation can be higher when ECoG-positive regions are left intact. The reason for the improvement of the language comprehension could be the preservation and the decompression of the auditory and Wernicke areas. However, ECoG can be identified only in the region of the functional cortex, not white matter. Therefore, awake craniotomy and/or cortico-cortical evoked potential (CCEP) must be considered to reveal and preserve the language network (27).

It is noteworthy that passive language mapping was successfully performed under general anesthesia, and language function preservation was achieved postoperatively in the present case. Pre and postoperative language functions were evaluated using the Standard Language Test of Aphasia (SLTA) which is widely administered in Japan (28). The SLTA is a comprehensive Japanese language test battery including 26 subtests for language comprehension, speaking, reading comprehension, writing, and calculation abilities.

The influence of general anesthesia should be considered when passive functional mapping under general anesthesia is done. Suzuki et al. (29) reported that the amplitudes of corticocortical evoked potential were increased in accordance with BIS level, and there were statistically significant differences between the awake (>80 BIS level) and anesthesia (<65 BIS level) states. However, to our best knowledge, there were no articles about a correlation between passive functional mapping and the depth of general anesthesia. We speculated that functional language mapping can be affected by the depth of anesthesia, therefore, anesthesia was maintained with a high BIS level in the present case. Further, studies about the influences of anesthesia on passive functional mapping are necessary to evaluate the intraoperative passive functional mapping accurately.

A limitation of this case report is that we do not know how the sensitivity and specificity of ECoG compared with ECS were in this case because ECS was not performed. We presume that passive language mapping will be widely applicable for the detection of brain function with the least stress for patients. We believe that real-time passive language mapping can be one of the most powerful tools to detect the region of the receptive-language function in brain surgery, especially for those who are not able to tolerate awake craniotomy.

## Conclusion

We performed real-time CortiQ-based passive language mapping under general anesthesia for an elderly patient with left temporal glioma. The patient's language function was well-preserved postoperatively. Further, studies are necessary to investigate the efficacy of intraoperative passive language mapping.

## Data Availability Statement

The original contributions presented in the study are included in the article/Supplementary Material, further inquiries can be directed to the corresponding author/s.

## Ethics Statement

Ethical review and approval was not required for the study on human participants in accordance with the local legislation and institutional requirements. Written informed consent to participate in this study was provided by the participants' legal guardian/next of kin. Written informed consent was obtained from the individual for the publication of any potentially identifiable images or data included in this article.

## Author Contributions

KK, TM, TK, and SK contributed conception and design of the study. KK wrote the first draft of the manuscript. TM, TK, and SK critically revised the final manuscript draft. All authors contributed to manuscript revision, read and approved the submitted version.

## Conflict of Interest

The authors declare that the research was conducted in the absence of any commercial or financial relationships that could be construed as a potential conflict of interest.

## Publisher's Note

All claims expressed in this article are solely those of the authors and do not necessarily represent those of their affiliated organizations, or those of the publisher, the editors and the reviewers. Any product that may be evaluated in this article, or claim that may be made by its manufacturer, is not guaranteed or endorsed by the publisher.
