# Supplementary material for: The Efficacy of Intraoperative Passive Language Mapping for Glioma Surgery: A Case Report
Source: Front Neurol. 2021 Aug 2;12:652401. doi: 10.3389/fneur.2021.652401 (PMC8364957; doi:10.3389/fneur.2021.652401)
Supplement: Supplementary file 2 [file Data_Sheet_1.docx]

The efficacy of intraoperative passive language mapping for glioma surgery: a case report

Authors

Kohei Kanaya, MD, PhD^1, 2^

Takumi Mitsuhashi, MD, PhD^3^

Takafumi Kiuchi, MD^2^

Sumio Kobayashi, MD, PhD^2^

^1^Department of Neurosurgery, Shinshu University School of Medicine, Matsumoto, Japan

^2^ Department of Neurosurgery, Iida Municipal Hospital, Iida, Nagano, Japan

^3^ Department of Neurosurgery, Juntendo University, Bunkyo-ku, Tokyo, Japan.

**1. Supplementary Video S1 legend.**

**2. Supplementary methods.**

**3. Supplementary references.**

**4. Supplementary Figure S1 legend.**

Video S1. The spatiotemporal dynamics of listening event-related cortical activation/deactivation under general anesthesia.

Circle size and color demonstrate the percent change of high-gamma activity in a given channel relative to the baseline period (i.e., between -600 and -200 ms relative to stimulus onset).

**Supplementary methods**

**Visualization of the dynamics of neuronal modulation during listening**

The goal of this analysis was to visualize listening event-related cortical modulation. The word/silence, sound/silence, and word/sound tasks each consisted of an 800 ms baseline period and an 800 ms stimulus period. We performed each task 60 times during the active phase. We quantified the event-related cortical modulation using a time-frequency analysis similar to those reported previously (Mitsuhashi et al., 2020,2021). The Morlet wavelet method, as implemented in the FieldTrip toolbox (http://www.fieldtriptoolbox.org), transformed electrocorticography signals into time-frequency bins (2 Hz frequency bins; [frequency divided by seven] cycles for each frequency; sliding in 10 ms steps) within a period from -100 to +800 ms with a bandpass of 60-90 and 110-140 Hz. We computed the percent change of amplitude relative to that during the 200-800 ms pre-stimulus baseline period for each frequency bin and averaged across 60 stimuli. We animated the percent change in high-gamma (60-90 and 110-140 Hz) amplitude at each electrode site and each moment on the intraoperative photograph of the cortical surface.

**Supplementary references**

Mitsuhashi T, Sonoda M, Iwaki H, Luat AF, Sood S, Asano E. Effects of depth electrode montage and single-pulse electrical stimulation sites on neuronal responses and effective connectivity. *Clin Neurophysiol* (2020) 131:2781-92. doi: 10.1016/j.clinph.2020.09.010.

Mitsuhashi T, Sonoda M, Jeong JW, Silverstein BH, Iwaki H, Luat AF, et al. Four-dimensional tractography animates propagations of neural activation via distinct interhemispheric pathways. *Clin Neurophysiol* (2021) 132:520-9. doi: 10.1016/j.clinph.2020.11.030.

**Figure S1: The changes of high-gamma amplitude in response to stimuli.**

High-gamma amplitude changes in (A) the channel responding to stimuli (channel #18) and (B) the channel not responding (channel #8). Green line: word/sound task. Blue line: sound/silence task. Red line: word/silence task. Shaded areas: 95% confidence intervals for responses to 60 stimuli.
