# Supplementary figures and images for: The Efficacy of Intraoperative Passive Language Mapping for Glioma Surgery: A Case Report
Source: Front Neurol. 2021 Aug 2;12:652401. doi: 10.3389/fneur.2021.652401 (PMC8364957; doi:10.3389/fneur.2021.652401)

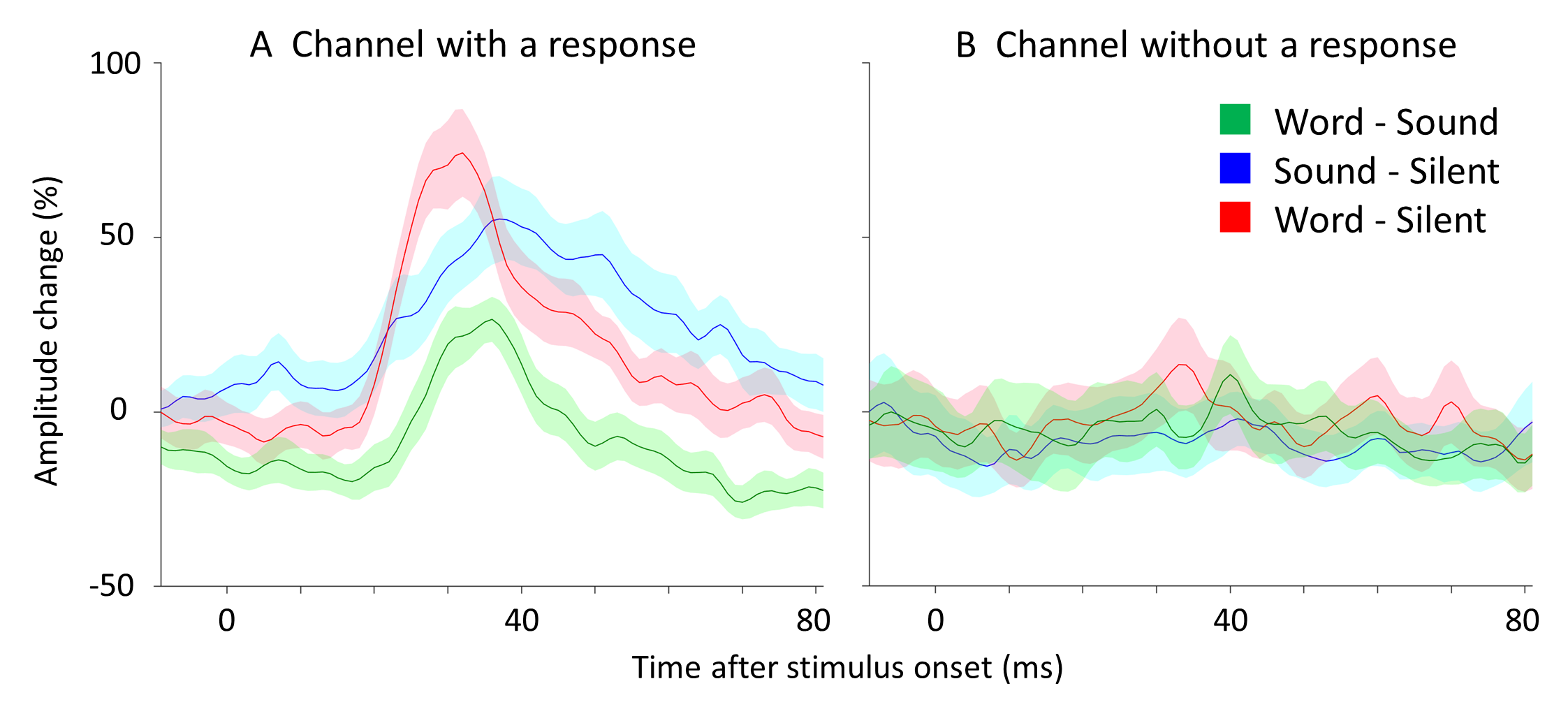

Supplement: Supplementary file 4 [file Image_1.TIF]
